# Biomonitoring of Perfluorinated Compounds in Children and Adults Exposed to Perfluorooctanoate-Contaminated Drinking Water

**DOI:** 10.1289/ehp.11064

**Published:** 2008-02-20

**Authors:** Jürgen Hölzer, Oliver Midasch, Knut Rauchfuss, Martin Kraft, Rolf Reupert, Jürgen Angerer, Peter Kleeschulte, Nina Marschall, Michael Wilhelm

**Affiliations:** 1 Department of Hygiene, Social and Environmental Medicine, Ruhr-University Bochum, Germany; 2 Institute and Outpatient Clinic of Occupational, Social and Environmental Medicine, University Erlangen-Nuremberg, Germany; 3 North Rhine-Westphalia State Agency for Nature, Environment and Consumer Protection, Recklinghausen, Germany; 4 Ministry of Environment and Conservation, Agriculture and Consumer Protection, North Rhine–Westphalia, Düsseldorf, Germany; 5 Public Health Department of the Hochsauerlandkreis, Meschede, Germany

**Keywords:** adults, children, drinking water, PFBS, PFC exposure, PFHxS, PFOA, PFOS

## Abstract

**Objective:**

40,000 residents in Arnsberg, Germany, had been exposed to drinking water contaminated with perfluorinated compounds (PFCs). Internal exposure of the residents of Arnsberg to six PFCs was assessed in comparison with reference areas.

**Design and participants:**

One hundred seventy children (5–6 years of age), 317 mothers (23–49 years), and 204 men (18–69 years) took part in the cross-sectional study.

**Measurements:**

Individual consumption of drinking water and personal characteristics were assessed by questionnaire and interview. Perfluorooctanoate (PFOA), perfluorooctanesulfonate (PFOS), perfluorohexanoate, perfluorohexanesulfonate (PFHxS), perfluoropentanoate, and perfluorobutanesulfonate (PFBS) in blood plasma and PFOA/PFOS in drinking water samples were measured by solid-phase extraction, high-performance liquid chromatrography, and tandem mass spectrometry detection.

**Results:**

Of the various PFCs, PFOA was the main compound found in drinking water (500–640 ng/L). PFOA levels in blood plasma of residents living in Arnsberg were 4.5–8.3 times higher than those for the reference population (arithmetic means Arnsberg/controls: children 24.6/5.2 μg/L, mothers 26.7/3.2 μg/L, men 28.5/6.4 μg/L). Consumption of tap water at home was a significant predictor of PFOA blood concentrations in Arnsberg. PFHxS concentrations were significantly increased in Arnsberg compared with controls (*p* < 0.05). PFBS was detected in 33% of the children, 4% of the women, and 13% of the men in Arnsberg compared with 5%, 0.7%, and 3%, respectively, in the reference areas (*p* < 0.05). Regression analysis showed that age and male sex were significant predictors of PFOS, PFOA, and PFHxS; associations of other regressors (diet, body mass index) varied among PFCs.

**Conclusions:**

PFC concentrations in blood plasma of children and adults exposed to PFC-contaminated drinking water were increased 4- to 8-fold compared with controls.

Perfluorinated compounds (PFCs) are widely distributed in the environment. Particularly, perfluorooctanesulfonate (PFOS) and perfluorooctanoate (PFOA) persist in humans and the environment. PFCs have been identified in surface water in the surroundings of fluorochemical production sites (Hansen et al. 2002), surface water from the Great Lakes (USA) (Sinclair et al. 2006) and oceans (Yamashita et al. 2005), wildlife (Giesy and Kannan 2001), and even in biota from remote regions (De Silva and Mabury 2004).

Human biomonitoring has proved to be a valuable tool to assess historical exposure to PFCs (Butenhoff et al. 2006). PFOS and PFOA have been detected in human blood samples of occupational (Olsen et al. 2003, 2007) and general populations. Data from the National Health and Nutrition Examination Survey (NHANES) 2003–2004 have been reported by Calafat et al. (2007). The authors observed reductions in the PFOA, PFOS, and perfluorohexanesulfonate (PFHxS) serum concentrations compared with NHANES 1999–2000. In a study comparing different countries, the highest PFOS concentrations were observed in blood plasma samples collected from the United States and Poland (> 30 μg/L); moderate in Korea, Belgium, Malaysia, Brazil, Italy, and Colombia (3–29 μg/L); and lowest in India (< 3 μg/L) (Kannan et al. 2004). In Germany recently, 12.2 μg PFOS/L and 5.3 μg PFOA/L (median values) were found in nonoccupationally exposed volunteers (14–67 years of age) living in the southern part of Bavaria, Germany (Fromme et al. 2007a). Midasch et al. (2006) reported 22.3 μg PFOS/L and 6.8 μg PFOA/L (median values) in 105 German nonsmokers. Human toxicology for PFOA and PFOS has also been reviewed recently [Kennedy et al. 2004; Kudo and Kawashima 2003; Lau et al. 2007; Organisation for Economic Co-operation and Development (OECD) 2002].

Despite the well-documented background exposure of the general population to PFCs, the main sources are not well known. Dietary intake seems to be the main source of exposure of the general population to PFOS and PFOA (Fromme et al. 2007b). To the best of our knowledge, a specific contaminated source has been described only in West Virginia (USA). In 2006, Emmett et al. published their investigations on PFOA contamination of drinking water in the surroundings of a fluoropolymer production site in Washington, West Virginia. The public water supply of Little Hocking, Ohio, draws water from wells across the Ohio River and has been contaminated by the highest PFOA concentrations reported in drinking water of public water supplies (mean, 3,550 ng/L; range, 1,500–7,200 ng/L) (Emmett et al. 2006a). Recently, a retrospective exposure assessment of the community was published (Paustenbach et al. 2007).

During their investigations into PFC concentrations of surface waters in Germany, Skutlarek et al. (2006) observed remarkably high PFOA concentrations not only in the rivers Ruhr (tributary of the Rhine, up to 177 ng/L) and Moehne (tributary of the Ruhr, up to 7,070 ng/L), but also in public water supplies, which use river water to produce drinking water by bank filtration or artificial recharge. The highest PFC concentration in drinking water, which was reported by Skutlarek et al., was 598 ng/L [sum of 519 ng PFOA, 5 ng PFOS, 11 ng perfluorobutanoic acid, 22 ng perfluorohexanoate (PFHxA), 5 ng perfluoropentanoic acid (PFPA), 23 ng perfluoroheptanoic acid, and 13 ng perfluorobutane sulfonate (PFBS) per liter] in Arnsberg-Neheim. The PFC concentrations in the river Moehne in Arnsberg-Neheim (sum value, 767 ng/L; PFOA, 647 ng/L) barely exceeded the PFC levels in corresponding drinking water (Skutlarek et al. 2006). In July 2006, the waterworks of Moehnebogen installed activated-charcoal filters, which efficiently decreased PFC concentrations in drinking water.

The finding of increased PFC concentrations in surface and drinking waters initiated an extensive environmental monitoring program. Meanwhile, systematic analyses of soil, ground, surface, and drinking water have been performed to identify sources and to assess human exposure to PFCs (Wilhelm et al., in press). Immediately after the increased PFOA levels were observed, the German Drinking Water Commission (DWC) of the German Ministry of Health at the Federal Environment Agency established guide values for human health protection (DWC 2006; Wilhelm et al., in press). As a result, local health authorities recommended that residents in parts of Arnsberg not use the drinking water for preparation of baby food, and advised pregnant women to avoid regular intake of such water. PFC-contaminated (> 500 ng PFOA/L) drinking water was supplied to approximately 40,000 residents (Kleeschulte et al. 2007). No data are available on PFC levels in drinking water in Arnsberg before May 2006.

By tracking the PFC contamination in the upper reaches of the river Moehne, the drainage of agricultural land was identified as the main source of contamination (Skutlarek et al. 2006). Based on the results of the extensive environmental monitoring program, federal health authorities concluded that PFC contamination of agricultural land occurred by the widespread use of soil conditioner, which had been mingled with industrial waste. Apparently PFC concentrations varied considerably between batches of material: Kraft et al. (2007) reported 650 μg PFOA and 8,600 μg PFOS/kg dry weight.

Between 2000 and 2006, farmers disseminated 27,700 tons of soil conditioner on almost 800 agricultural land sites in the catchment areas of the rivers Moehne and Upper-Ruhr. Overall, 53,000 tons of the mixture were applied to > 1,300 areas in North Rhine–Westphalia (Delschen et al. 2007). All areas in the catchment area of the rivers Moehne and Upper-Ruhr where the particular soil conditioner was disseminated were screened for PFC contamination by analyzing surface soil samples. In most soil samples, no or low PFC concentrations were observed, whereas in few cases high PFC concentrations were found (District Government Arnsberg 2007). The most relevant PFC pollution (6,300 μg PFOS + PFOA/kg) with respect to drinking-water contamination was observed in a 10 hectares–wide agricultural area in Brilon-Scharfenberg (Bergmann et al., in press). The area in Brilon-Scharfenberg drains into the Steinbecke and Bermecke, small creeks that are tributaries of the river Moehne. After installation of a special drainage around the area with water treatment by activated-charcoal filtering, the PFC concentrations in the river Moehne decreased distinctly.

We report here on the results of a biomonitoring study performed between September and November 2006. The study was designed to determine the concentrations of PFCs in blood plasma of subjects affected by PFOA contamination of public water supplies compared with reference areas; explore the association between personal drinking-water consumption and the concentrations of PFCs in blood plasma; and explore the association between diet (consumption of locally grown fruits and vegetables, locally caught fish) and the concentrations of PFCs in blood plasma.

## Study Design, Population, and Methods

The cross-sectional study was performed between September and November 2006 in Arnsberg, Siegen, and Brilon. Arnsberg (75,000 inhabitants) and Brilon (25,000 inhabitants) are located in the eastern part of North Rhine–Westphalia, known as “Hochsauerland,” which is an important recreation area for the state. Siegen (100,000 inhabitants) lies in the south Westphalian part of North Rhine–Westphalia.

**Figure 1:**
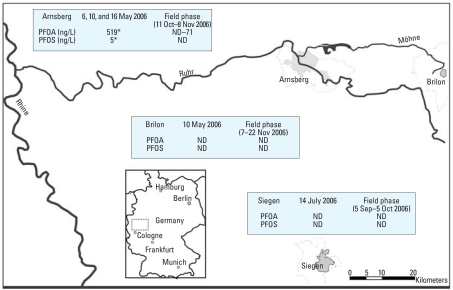
Location of study and reference areas and PFOA and PFOS concentrations in drinking water. All analyses were performed by North Rhine–Westphalia State Agency for Nature, Environment and Consumer Protection except those marked by an asterisk, which were analyzed and reported by Skutlarek et al. (2006). ND, not detected. Tap-water samples were collected from the kitchen at the homes of the residents; analyses were performed for every day that blood samples were taken during the study period. In July 2006, waterworks installed activated charcoal filters. LOD = 10 ng/L.

Study areas were predefined on the basis of the water supply system structure and PFC water analyses. Four (of 15) boroughs in Arnsberg had been constantly supplied by the Moehnebogen waterworks with PFOA-contaminated drinking water: Neheim, Huesten, Herdringen, and Bruchhausen. These boroughs were selected as the target area and are referred to here as “Arnsberg.” PFOA or PFOS could not be detected in drinking-water samples in the reference areas Brilon and Siegen (Figure 1).

### Study population

Male adults were recruited from age-stratified random samples of the male population 18–69 years of age in Arnsberg (*n* = 14,167) and Brilon (*n* = 8,608), respectively. Home addresses were provided by the registration of address office. Randomly selected subjects (Arnsberg, 527; Brilon, 500) were informed in writing and subsequently contacted by telephone for a short interview to assess personal consumption of drinking water. At least three attempts were made to contact the addressed persons. Contact was successful for 234 men in Arnsberg and 296 in Brilon. Of those men contacted, 199 (85%) in Arnsberg and 200 (68%) in Brilon answered the questionnaire and consented to participate. Based on the evaluation of the questionnaire data, 100 men with the highest self-reported tap water consumption were selected to participate in Arnsberg and Brilon, respectively.

For the recruitment of mothers and children, we chose a different approach. Parents of all school beginners in the affected districts (*n* = 364) and in the reference area (*n* = 417) were informed in writing and asked to participate. Contact was successful with 217 mothers (60%) in Arnsberg and 262 (63%) in Siegen. Of those contacted, 164 (76%) in Arnsberg and 153 (58%) in Siegen finally participated.

Informed consent was obtained from the participants and the parents of the children before the study. The study was approved by the ethical commission of the Ruhr-University of Bochum and was conducted in accordance with the ethical principles for medical research involving human subjects as defined by the Helsinki Declaration.

### Questionnaire

A questionnaire for self-completion was sent to the study participants to obtain information on characteristics such as height, body weight, school education, smoking habits, exposure to environmental tobacco smoke, and environmental or occupational exposures to PFCs. On the date the blood samples were taken, trained interviewers administered a standardized questionnaire on drinking-water consumption and diet. Drinking-water consumption was itemized as follows: pure water, tea or coffee, preparations with juice concentrates, and soups. Consumption at home and at the workplace (or at kindergarten for the children) were recorded separately. To account for temporal changes in consumer behavior, actual as well as past drinking-water consumption was recorded. Parents answered questions for their children.

### Sampling of blood and drinking water

Blood collection began on 4 September and ended 22 November 2006. Standard materials for venipuncture and blood sampling were used. Blood samples were processed on the same day. Plasma samples were stored at −20°C and transported frozen to the analytical toxicologic laboratories in Erlangen, Germany. All materials were tested for PFC contamination before the start of the study. No contamination was found.

Tap-water samples were collected from the kitchen in the homes of all residents. Sampling vessels (polypropylene, 50 mL; Greiner Bio-One, Frickenhausen, Germany) were thoroughly rinsed with methanol before use. On the day the blood samples were drawn, tap-water samples were taken by the study participants following written instructions. Water samples were frozen, stored at −20°C, and transported to the laboratories for further processing. One random water sample per day was analyzed.

Both laboratories (for blood and water analyses) were blinded with respect to the origin of the samples and performed the analyses after the end of the field phase.

### Analysis of blood plasma

Human plasma samples were analyzed for PFOS, PFHxS, PFBS, PFOA, PFHxA, and PFPA. The analytes were extracted from interfering matrix compounds by solid-phase extraction (Taniyasu et al. 2005). After elution, the analytes were chromatographically separated by high performance liquid chromatography (HPLC) and detected by tandem mass spectrometry. Calibration was performed using standard solutions prepared in bovine serum which were treated in the same manner as the human plasma samples analyzed. As internal standards, ^13^C-labeled analogs of PFOS, PFOA, and PFHxA were used.

Five hundred microliters of the plasma aliquot was spiked with 50 μL internal standard solution [1,2,3,4-^13^C_4_-PFOS and 1,2-^13^C_2_-PFHxA (Wellington Laboratories, Guelph, Ontario, Canada) and 1,2-^13^C_2_-PFOA (Medical Laboratory, Bremen, Germany); concentrations were 250 μg/L in acetonitrile]. To precipitate plasma proteins, 2 mL formic acid (50% aq., vol/vol) were added, and the sample was sonicated for 15 min. After centrifugation, the supernatant was used for solid-phase extraction on a Waters Oasis WAX cartridge (Waters GmbH, Eschborn, Germany), which was preconditioned with 2 mL methanol and 2 mL water (Taniyasu et al. 2005). After drawing the sample through, the cartridge was rinsed with 2 mL methanol solution (40% aq., vol/vol). Elution was obtained by 1 mL 2% NH_4_OH in methanol. The eluate was dried under a stream of nitrogen to a volume of approximately 100 μL and diluted afterward with 75 μL ammonium acetate solution (20 mmol aq.) in methanol (25/75, vol/vol). Chromatographic separation was performed on an Agilent Zorbax Eclipse XDB-C8 column (GGA GmbH, Moers, Germany). For detection, an Applied Biosystems API 2000 triple quadrupole mass spectrometer (Foster City, CA, USA) was operated in negative electrospray ionization (ESI) and multiple reaction monitoring (MRM) modes. In each analytical batch, one reagent blank (water instead of plasma), one serum blank, the calibration standards [all standard substances purchased from Sigma-Aldrich (Taufkirchen, Germany), concentration of the analytes 2.5 to 50 μg/L, prepared in bovine serum], and one quality control sample were analyzed parallel to the human plasma samples. Reagent blanks were subtracted from the obtained plasma concentrations. Limits of detection were estimated to be 0.1 μg/L for PFOS, PFHxS, PFBS, and PFOA, and 1.0 μg/L for PFHxA and PFPA based on a 3-fold signal-to-noise ratio. Quality control of the analytical results was carried out as stipulated in the guidelines of the German Medical Association for quality assurance of quantitative analyses in laboratory medicine (Bundesaerztekammer 2001).

Within-series imprecision was determined by analyzing a pooled human plasma sample spiked with PFHxS, PFBS, PFHxA, and PFPA (~5 μg/L each). For PFOS and PFOA the native concentrations were measured (~ 9.5 and 5.5 μg/L, respectively). The coefficients of variation (CVs) were between 4.4 and 10.9% at 10 repetitions. The between-day imprecision was determined by analyzing the same material 49 times on different days (2–3 times/day). The CVs thereby ranged from 6.9 to 12.7%. Depending on the analyte, mean relative recoveries were between 101.9 and 122.0% at a spiked concentration of 10 μg/L using five different plasma samples.

### Analysis of drinking water

Forty milliliters of the water sample was spiked with internal standard substances (^13^C_4_-PFOA, ^13^C_4_-PFOS). Solid-phase extraction was performed on a modified polymer cartridge (Isolute ENV+; 25 mg; Separtis GmbH, Grenzach-Wyhlen, Germany), which was preconditioned with 2 mL methanol and 2 mL water; 10 mL of the prepared water sample was passed through. After a washing step with 2 mL water, the adsorbent material was dried in the airflow. Analytes were eluted with 1.5 mL methanol and reduced to dryness under nitrogen gas flow at 40°C and then solved in 1 mL of a mixture of water/methanol (8/2, vol/vol).

PFCs were separated by HPLC by gradient elution on an RP-C18 phase using an acetate buffer solution in methanol and water. Compounds were detected with a tandem quadrupole mass spectrometer employing ESI in negative mode and MRM.

We identified PFCs by the retention time and the intensity coefficient of two mass transitions for each compound. Calibration was performed with internal standard evaluation. The linear concentration range was 0.1–2.5 ng/mL. The reference standard solutions (linear compounds only) were purchased from Wellington Laboratories. The signal of the isomer part was calculated on the base of the response of the linear compound. The results are given as the sum of the concentration of all identified linear and branched isomers. The limit of detection (LOD) was 10 ng/L (signal to noise > 10:1, peak-to-peak mode).

We analyzed the blank of the whole procedure within every sample series; in all series it was < 2 ng/L for PFOA and PFOS. We determined the measurement uncertainty by standard addition method; it was < 20% at a concentration level of 100 ng/L. Typical method recoveries were 98.5 ± 1.9% (*n* = 6) for PFOA and 85.1 ± 4.1% (*n* = 6) for PFOS, calculated by external standardization. The method for PFC analysis in drinking water is described in International Organization for Standardization (ISO)/CD 25101 (ISO 2008).

### Data and statistical methods

For PFC concentrations below the LOD, an imputed value equal to one-half the LOD was used. Minima, lower quartiles, medians, upper quartiles, maxima, and geometric means (with 95% confidence intervals) of the PFCs in Arnsberg and in controls are presented in box and whisker plots.

Geometric mean levels are presented because most of the distributions are not normal but “skewed to the right,” i.e., higher values. We calculated Spearman rank correlation coefficients (*r_S_*) to evaluate the closeness of relationship between two continuous variables.

We performed multivariate analyses through linear regression models. Dependent variables were log_10_(PFOA, PFOS, PFHxS) concentrations in blood plasma (nanograms per liter). Regressors were chosen based on the study hypothesis (region, consumption of drinking water, locally caught fish, locally grown fruits and vegetables), other studies (age, sex), and the results of bivariate calculations [body mass index (BMI)]. Additionally, we computed standardized parameter estimates. For adults, all variables were included in the respective model. For children, a stepwise procedure (SAS Institute Inc. 2004) was used to identify significant regressors. All data were analyzed using statistical software package SAS version 9.1.3 (SAS Institute Inc., Cary, NC, USA).

## Results

One hundred ninety-nine men in Arnsberg (85% of all men who were successfully contacted) and 200 men (68%) in Brilon answered the questionnaire and gave their consent to participate. One hundred three blood samples were drawn from men in Brilon and 101 in Arnsberg.

One hundred seventy-four mother–child pairs (80%) in Arnsberg and 182 (70%) in Siegen gave their consent to participate. One hundred fifty-three blood samples were drawn from mothers and 80 from children in Siegen, and 164 and 90 in Arnsberg (Table 1).

Age, height, body weight and sex were comparable between Arnsberg and the reference groups in Siegen and Brilon (Table 2).

PFOA and PFOS were not detected in drinking-water samples from reference areas Siegen and Brilon (Figure 1). After installation of activated charcoal filters in the waterworks, PFOA concentrations in Arnsberg were significantly reduced. However, during the study period, filtration performance declined and PFOA concentrations in tap-water samples increased from below the LOD to 71 ng/L.

All measured PFOA and PFOS concentrations in blood plasma exceeded the LOD (0.1 μg/L). PFPA and PFHxA were not detected in any of the samples (LOD: 1 μg/L).

PFOA levels of children and adults living in Arnsberg were 4.5–8.3 times higher compared with the reference population (ratios based on arithmetic means: children 24.6/5.2 μg/L, mothers 26.7/3.2 μg/L, men 28.5/6.4 μg/L, Table 3).

PFOS concentrations were not significantly different in the groups of the men and the children (*p* = 0.30 for both groups). However, in the blood of mothers in Arnsberg, 12% higher PFOS concentrations were observed compared with the control area in Siegen (*p* < 0.05, Table 3).

PFHxS concentrations were significantly increased in Arnsberg compared with the respective reference areas (*p* < 0.05, Table 3). In Arnsberg, the PFHxS concentrations were 14% (men), 53% (children), and 80% (mothers, geometric mean) higher.

PFBS was detected in 33% of the blood samples of children, 4% of the women, and 13% of the men in Arnsberg compared with 5%, 0.7%, and 3%, respectively, in the reference areas (data not shown). The proportion of concentrations above the LOD (0.1 μg/L) was higher in Arnsberg compared with the reference areas (*p* < 0.05, Fisher’s exact test). The maximum PFBS concentration detected in a blood sample was 0.46 μg/L. Ninety-fifth percentiles of PFBS concentrations observed in children in Arnsberg were 0.2 μg/L, for women, < LOD, and for men, 0.2 μg/L compared with 0.1 μg/L, < LOD, and < LOD, respectively, in the reference areas.

PFOA levels were associated with increased PFOS and PFHxS concentrations. This effect was most pronounced in the reference areas [*r_S_* = 0.54/0.77 (children), 0.62/0.57 (mothers), 0.53/0.49 (men); *p* < 0.01]. In Arnsberg, the associations were weaker [*r_S_* = 0.32/0.37 (children), not significant/0.50 (mothers), 0.27/0.49 (men); *p* < 0.01] (Table 4).

Statistically significant (*p* < 0.01) correlations existed between age and PFOA (mothers and men in both areas), PFOS (men in both areas, mothers in the reference area), and PFHxS (men in both areas, mothers in Arnsberg) (Table 4).

**Figure 2:**
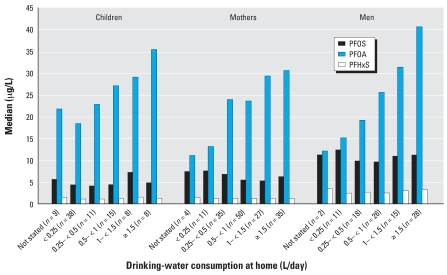
Self-assessed consumption of drinking water at home and PFOA, PFOS, and PFHxS concentrations in blood plasma (median) in Arnsberg.

Consumption of drinking (tap) water was clearly associated with raised PFOA concentrations in blood plasma in Arnsberg (Figure 2). The more tap water was consumed at home, the higher PFOA concentrations we observed in blood plasma. Study participants who reported drinking > 1.5 L tap water daily had about 2-fold increased PFOA plasma levels compared with those who reported drinking < 0.25 L/day [median plasma concentrations (μg PFOA/L): children, 35 (*n* = 8)/18 (*n* = 38); women, 31 (*n* = 35)/13 (*n* = 11); men, 41 (*n* = 28)/15 (*n* = 11)].

Table 5 presents characteristics of linear regression models of PFC concentrations in plasma samples of adults. Consumption of drinking water at home, locally grown fruits and vegetables, fish caught from local lakes, age, sex, BMI, and area (Arnsberg, reference area) were chosen as independent variables. The proportions of variance of PFOA, PFOS, or PFHxS, which were predictable from the regressor variables, varied between 0.27 and 0.79 (adjusted *R*^2^). PFOA levels in adults were associated with consumption of drinking water at home (*p* < 0.01), locally grown fruits and vegetables (*p* = 0.059), age, male sex, region (all *p* < 0.01), and inversely with BMI (*p* = 0.05). Age, male sex, region, and consumption of locally caught fish (all *p* < 0.01), and (inversely) BMI (*p* = 0.02) were predictors of PFOS concentrations in adults. PFHxS was associated with consumption of drinking water (*p* = 0.066), male sex, age, and study area (all *p* < 0.01).

In children, PFOA concentrations in blood plasma were associated with drinking water consumption at home and region (*p* < 0.01, adjusted *R* ^2^ = 0.75). Region (*p* < 0.01) and male sex (*p* = 0.07) were regressors for internal exposure of children to PFHxS (adjusted *R* ^2^ = 0.16), whereas none of the independent variables mentioned was significantly associated with PFOS concentrations in blood plasma of children. No statistically significant association was found between PFOA or PFOS and smoking status (adults) or exposure to environmental tobacco smoke (children, data not shown).

## Discussion

Several limitations must be considered with regard to eligible exact exposure quantification. Local authorities informed the public about the contamination of the drinking water in May 2006. On receiving this information, > 50% of the mother–child pairs and > 25% of the men in Arnsberg reduced their personal drinking-water consumption, for example by using bottled water. Blood samples were collected in October 2006, that is, 5 months after publication of the first reports on the PFOA contamination and 3 months after distinct reductions in the exposure situation had taken place. However, PFC half-lives in humans presumably are long and probably unaffected by short-term changes. Based on a 5-year follow-up of 26 retired fluorochemical production workers, Olsen et al. (2007) recently calculated the geometric mean half-lives of human serum elimination of 4.8 years for PFOS, 7.3 years for PFHxS, and 3.5 years for PFOA.

We do not know how many years the PFOA contamination of the drinking water had already lasted and whether the PFOA levels in drinking water might have been even higher during the most recent years. Questionnaire data on drinking-water-consumption behavior before May 2006 are the best data available and therefore were chosen as a proxy measurement for exposure. Tap-water consumption assessed by interview compared fairly well with standard exposure factors published by the National Center for Environmental Assessment (NCEA), Office of Research and Development of the U.S. Environmental Protection Agency. Interview-derived mean tap water consumption rates of children (mothers, men) were 0.7 (1.2, 1.4) L/day; NCEA-recommended drinking water intakes rates were 0.7 L/day for children 1–10 years of age and 1.4 L/day for adults (NCEA 1997).

We assessed drinking-water use by an interview based on a structured questionnaire. Such information is subject to over- and underreporting, particularly in this case, because the participants in Arnsberg may have been especially aware of that route of exposure because of the media coverage.

The statistical association between consumption of locally caught fish and PFOS blood concentrations seems plausible. Several reports on elevated PFOS concentrations in fish and on the biomagnification potential of PFCs have been published recently. Nakata et al. (2006) investigated PFC in sediments and aquatic organisms of the Ariake Sea, Japan. They reported PFOS as the dominant contaminant and observed elevated concentrations in higher trophic level species. Sinclair et al. (2006) found average concentrations of PFOS in fish that were 8,850-fold greater than those in surface waters from New York State. Haukas et al. (2007) reported bio-magnification factors > 1 for PFHxS, perfluorononanoic acid, PFOS, and the sum of polyfluorinated alkyl substances in selected species from the Barents Sea food web. PFC concentrations up to 425 μg PFOS and up to 12 μg PFOA/kg wet weight were observed in fish caught in the upper Moehne and in the Lake Moehnesee (Wilhelm et al., in press).

The first human biomonitoring study that assessed drinking water as a source of exposure to PFCs was carried out in Little Hocking, Ohio, (Emmett et al. 2006a, 2006b). During the investigation period from 2002 to 2005, a mean concentration of 3,500 ng PFOA/L (range, 1,500–7,200 ng/L) in drinking water was reported. This level is about 7-fold higher than the highest concentrations measured in drinking water samples in Arnsberg in May 2006. PFOA concentrations in serum of the population in Little Hocking (people who were supplied by the Little Hocking water system only; arithmetic mean, 448 μg/L; *n* = 291) are about 16- to 18-fold increased compared with the mean plasma levels of the population in Arnsberg.

**Figure 3:**
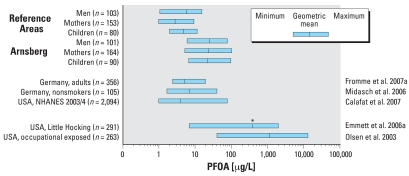
PFOA concentrations in human blood in Arnsberg and reference areas compared with national and international data. *Median instead of geometric mean.

Plasma and serum concentrations of PFOA, PFOS, and PFHxS have been found comparable (Ehresman et al. 2007). Most national and international studies use these biological matrices. The PFOA concentrations in our reference groups compare well with data from other actual German studies and to the NHANES 2003–2004 data (Calafat et al. 2007; Fromme et al. 2007a; Midasch et al. 2006) (Figure 3).

## Conclusion

This cross-sectional study demonstrated distinctly elevated PFC concentrations in blood plasma of children and adults exposed to PFC-contaminated drinking water in an environmental setting. Because of the origin of the contamination—the disposal of PFC-contaminated soil conditioner on agricultural areas that drain into a drinking water reservoir—precise data about the PFC concentrations in drinking water have been available since the first detection of the PFC contamination in May 2006.

Of the various PFCs, PFOA was the main compound found in drinking water (500–640 ng/L). PFOA levels in blood plasma of residents living in Arnsberg were 4.5–8.3 times higher than in the reference population. In this respect, our study provides affirmation to the publication of Emmett et al. (2006a) but on a much lower level of exposure (about one-seventeenth with regard to blood levels, about one-seventh concerning drinking-water concentrations). PFHxS concentrations were significantly increased in Arnsberg compared with controls (*p* < 0.05). PFBS was detected in 33% of the children, 4% of the women, and 13% of the men in Arnsberg compared with 5%, 0.7%, and 3%, respectively, in the reference areas (*p* < 0.05). PFOS concentrations were not significantly different in the groups of the men and the children (*p* = 0.30 for both groups). However, in the blood of mothers in Arnsberg, 12% higher PFOS concentrations were observed compared with the control area in Siegen (*p* < 0.05, Table 3). Age and male sex were significant predictors of PFOS, PFOA, and PFHxS; associations of other regressors (diet, BMI) varied among PFCs.

## Figures and Tables

**Table 1:** Sampling, selection criteria, and response rates in Arnsberg and reference areas (Siegen and Brilon).

	Mother–child pairs^a^		Men (18–69 years)^b^	
Characteristic	Siegen	Arnsberg	Brilon	Arnsberg
Size of selected populations (no.)	417	364	8,608	14,167
No. of subjects				
Received the information letter	417	364	500	527
Were contacted successfully	262	217	296	234
Gave consent to participate	182	174	200	199
Refused consent	80	43	96	35
Filled in nonresponder questionnaire	51	16	58	16
Blood samples				
Mothers	153	164		
Children	80	90		
Men			103	101

a Sample included all mother–child (school beginners) pairs in selected districts.

b Age-stratified random sample.

**Table 2:** Characteristics of study groups in Arnsberg and reference areas (Siegen and Brilon).

	Children		Mothers		Men	
Characteristic	Siegen	Arnsberg	Siegen	Arnsberg	Brilon	Arnsberg
No.	80	90	153	164	103	101
Male:female (%)	57:43	54:46				
Age (years, mean)	5.7	5.8	35.4	36.2	47.9	47.1
Body weight (kg, mean)	20.9	21.8	68.2	69.8	86.4	85.4
Height (cm, mean)	116.7	118.2	166.7	167.8	179.4	181.1

**Table 3:** PFC concentrations in blood plasma of study populations in Arnsberg and reference areas (Brilon, Siegen).

	No.	No. < LOD	AM	SD	Min	75th	95th	Max	GM	95% CI
PFOA (μg/L)										
Children										
Siegen	80	0	5.2	2.1	2.0	6.5	9.1	11.5	4.8	4.4–5.2
Arnsberg	90	0	24.6	12.9	6.7	29.4	46.3	96.6	22.1	20.1–24.4
Mothers										
Siegen	153	0	3.2	1.5	0.7	4.1	5.9	9.2	2.8	2.6–3.1
Arnsberg	164	0	26.7	13.8	5.4	32.7	52.4	99.7	23.4	21.6–25.4
Men										
Brilon	103	0	6.4	2.8	1.1	8.3	10.9	15.3	5.8	5.3–6.3
Arnsberg	101	0	28.5	12.9	6.1	38.4	46.6	77.5	25.3	22.9–28.0
PFOS (μg/L)										
Children										
Siegen	80	0	5.2	3.4	1.6	6.1	11.5	26.2	4.6	4.1–5.1
Arnsberg	90	0	5.4	2.9	2.4	6.0	10.5	20.6	4.9	4.5–5.4
Mothers										
Siegen	153	0	6.2	6.2	1.0	7.3	13.5	70.7	5.2	4.7–5.6
Arnsberg	164	0	6.3	2.8	1.7	7.6	11.5	16.7	5.8	5.4–6.2
Men										
Brilon	103	0	12.4	11.5	1.7	14.0	26.4	92.5	9.7	8.5–11.0
Arnsberg	101	0	11.8	6.1	2.7	14.8	23.4	36.2	10.5	9.6–11.6
PFHxS (μg/L)										
Children										
Siegen	80	1	1.0	1.1	< LOD	1.0	2.2	9.1	0.8	0.7–0.9
Arnsberg	90	0	1.4	1.5	0.5	1.5	2.1	13.4	1.2	1.1–1.3
Mothers										
Siegen	153	2	0.7	0.4	< LOD	0.9	1.5	2.1	0.6	0.6–0.7
Arnsberg	164	2	1.2	0.6	< LOD	1.5	2.2	5.7	1.1	1.0–1.2
Men										
Brilon	103	0	2.4	1.0	0.7	3.0	4.3	5.4	2.2	2.0–2.4
Arnsberg	101	0	2.7	1.1	0.7	3.2	4.4	8.7	2.5	2.3–2.8

Abbreviations: AM, arithmetic mean; GM, geometric mean; Max, maximum; Min, minimum; 95% CI, 95% confidence interval of the geometric mean. 75th and 95th are percentiles. LOD = 0.1 μg/L.

**Table 4:** Spearman rank correlation coefficients for PFOA, PFOS, PFHxS, BMI, and age.

	PFOA	PFOS	PFHxS	BMI	Age
Men					
Reference area (*n* = 103)				**Arnsberg (*n*= 101)**	
PFOA		0.27**	0.49**	**0.09**	0.43**
PFOS	0.53**		0.48**	−0.26**	0.26**
PFHxS	0.49**	0.64**		**0.02**	0.31**
BMI	0.03	0.11	0.12		0.30**
Age	0.42**	0.40**	0.27**	0.30**	
Mothers					
Reference area (*n* = 153)				**Arnsberg (*n*= 164)**	
PFOA		**0.11**	0.50**	−0.19*	0.19*
PFOS	0.62**		0.43**	−**0.11**	**0.11**
PFHxS	0.57**	0.58**		**0.002**	0.23**
BMI	−0.10	−0.09	0.03		**0.04**
Age	0.27**	0.18*	0.09	0.001	
Children					
Reference area (*n* = 80)				**Arnsberg (*n*= 90)**	
PFOA		0.32**	0.37**	**0.02**	−**0.07**
PFOS	0.54**		0.56**	**0.07**	**0.02**
PFHxS	0.77**	0.68**		**0.03**	**0.10**
BMI	−0.16	−0.23#	−0.25*		−**0.10**
Age	0.18	0.21#	0.14	−0.08	

*Prob* > |*r*| under *H_0_*: Rho = 0.

* 0.05 > *p* ≥ 0.01.

** *p* < 0.01.

# 0.1 > *p* ≥ 0.05.

**Table 5:** Results of multiple regression analysis on PFOA , PFOS, and PFHxS concentrations in blood plasma samples of adults.

	log_10_[PFOA (ng/L)]			log_10_[PFOS (ng/L)]			log_10_[PFHxS (ng/L)]		
	β	95% CI	SE	β	95% CI	SE	β	95% CI	SE
Consumption of drinking water (L/day)	0.062**	0.040 to 0.083	0.12	−0.007	−0.030 to 0.016	−0.02	0.020#	−0.001 to 0.042	0.06
Consumption of locally grown fruits and vegetables (per week)	0.005#	0.0002 to 0.011	0.04	−0.0001	−0.006 to 0.006	−0.001	0.002	−0.003 to 0.008	0.02
Consumption of locally caught fish (per week)	0.059	−0.027 to 0.144	0.03	0.123**	0.030 to 0.216	0.10	0.037	−0.049 to 0.123	0.03
BMI (kg/m^2^)	−0.004*	−0.009 to 0.0003	−0.05	−0.006*	−0.010 to −0.001	−0.10	−0.001	−0.005 to 0.003	−0.01
Age (years)	0.007**	0.005 to 0.009	0.17	0.006**	0.004 to 0.008	0.25	0.005**	0.003 to 0.007	0.18
Male sex	0.074**	0.029 to 0.119	0.08	0.195**	0.147 to 0.244	0.36	0.384**	0.339 to 0.429	0.60
Region (Arnsberg)	0.801**	0.763 to 0.840	0.87	0.030	−0.012 to 0.072	0.06	0.159**	0.120 to 0.197	0.25

Abbreviations: β, parameter estimates; 95% CI, 95% confidence interval of the parameter estimate; SE, standardized estimate. Adjusted *R*^2^ = 0.79 ng/L for log_10_(PFOA), 0.27 for log_10_(PFOS), and 0.55 for log_10_(PFHxS).

* 0.01 ≤ *p* < 0.05.

** *p* < 0.01.

# 0.05 ≤ *p* < 0.1.
